# Japanese Society of Medical Oncology/Japan Society of Clinical Oncology/Japanese Society of Pediatric Hematology/Oncology-led clinical recommendations on the diagnosis and use of immunotherapy in patients with high tumor mutational burden tumors

**DOI:** 10.1007/s10147-023-02360-8

**Published:** 2023-06-10

**Authors:** Saori Mishima, Yoichi Naito, Kiwamu Akagi, Naomi Hayashi, Akira Hirasawa, Tomoro Hishiki, Ataru Igarashi, Masafumi Ikeda, Shigenori Kadowaki, Hiroaki Kajiyama, Motohiro Kato, Hirotsugu Kenmotsu, Yasuhiro Kodera, Keigo Komine, Takafumi Koyama, Osamu Maeda, Mitsuru Miyachi, Hiroshi Nishihara, Hiroyuki Nishiyama, Shouichi Ohga, Wataru Okamoto, Eiji Oki, Shigeru Ono, Masashi Sanada, Ikuo Sekine, Tadao Takano, Kayoko Tao, Keita Terashima, Katsuya Tsuchihara, Yasushi Yatabe, Takayuki Yoshino, Eishi Baba

**Affiliations:** 1 National Cancer Center Hospital East, Kashiwa, Japan; 2 Saitama Cancer Center, Saitama, Japan; 3 The Cancer Institute Hospital of Japanese Foundation for Cancer Research, Tokyo, Japan; 4 Okayama University, Okayama, Japan; 5 Chiba University, Chiba, Japan; 6 Yokohama City University School of Medicine, Yokohama, Japan; 7 Aichi Cancer Center, Aichi, Japan; 8 Nagoya University, Aichi, Japan; 9 The University of Tokyo, Tokyo, Japan; 10 Shizuoka Cancer Center, Shizuoka, Japan; 11 Nagoya University Hospital, Aichi, Japan; 12 Tohoku University, Miyagi, Japan; 13 National Cancer Center Hospital, Tokyo, Japan; 14 Kyoto Prefectural University of Medicine, Kyoto, Japan; 15 Keio University, Tokyo, Japan; 16 Tsukuba University, Ibaraki, Japan; 17 Kyushu University, Fukuoka, Japan; 18 Hiroshima University Hospital, Hiroshima, Japan; 19 Jichi Medical University, Tochigi, Japan; 20 National Hospital Organization Nagoya Medical Center, Aichi, Japan; 21 National Center for Child Health and Development, Tokyo, Japan

**Keywords:** Advanced solid tumor, Clinical practice guideline, Tumor mutation burden-high (TMB-H), Immunotherapy, PD-1/PD-L1 inhibitor

## Abstract

The development of novel antitumor agents and accompanying biomarkers has improved survival across several tumor types. Previously, we developed recommendations for tumor-agnostic treatments in patients with solid tumors with DNA mismatch repair deficient or neurotrophic receptor tyrosine kinase fusions. Recently, immune checkpoint inhibitors have shown efficacy in patient with tumor mutation burden-high (TMB-H) solid tumors and have been established as a third tumor-agnostic agent, making it necessary to develop the guideline prioritized for these patients. Clinical questions regarding medical care were formulated for patients with TMB-H advanced solid tumors. Relevant publications were searched by PubMed and Cochrane Database. Critical publications and conference reports were added manually. Systematic reviews were performed for each clinical question for the purpose of developing clinical recommendations. The committee members identified by Japan Society of Clinical Oncology (JSCO), Japanese Society of Medical Oncology (JSMO), and Japanese society of pediatric hematology/oncology (JSPHO) voted to determine the level of each recommendation considering the strength of evidence, expected risks and benefits to patients, and other related factors. Thereafter, a peer review by experts nominated from JSCO, JSMO, and JSPHO, and the public comments among all societies' members was done. The current guideline describes three clinical questions and seven recommendations for whom, when, and how TMB should be tested, and what is recommended for patients with TMB-H advanced solid tumors. In this guideline, the committee proposed seven recommendations for performing TMB testing properly to select patients who are likely to benefit from immunotherapy.

## Introduction

In the field of cancer drug therapy, treatment outcomes and prognosis have improved as effective novel drug therapies have emerged [1, 2]. At the same time, the development of biomarkers to identify groups in which efficacy is likely prior to treatment also has contributed to improvements in cancer treatment outcomes. Conventional cancer treatment has involved a multifaceted assessment that encompasses the pathological diagnosis of the disease and an evaluation of its stage, the benefits and disadvantages of treatment, and the preferences of the patient. In diagnosing the disease, identifying the primary tumor and determining the tissue type have yielded important information that has been key to establishing a treatment plan. Recent advances in molecular biology have elucidated a variety of biological characteristics of tumors, resulting in the clinical development and regulatory approval of tumor-agnostic drugs that transcend the organic characteristics of the disease.

An anti-programmed cell death protein 1 (PD-1) antibody drug, pembrolizumab, for advanced/recurrent deficient DNA mismatch repair (dMMR) solid cancers and tropomyosin receptor kinase (TRK) inhibitors against neurotrophic receptor tyrosine kinase (NTRK) fusion gene-positive advanced solid cancers were approved in tumor-agnostic therapy [3]. Moreover, the efficacy of pembrolizumab against tumor mutation burden-high (TMB-H) solid tumors was demonstrated, and the US Food and Drug Administration (FDA) approved it in 2020 [3]. In Japan, pembrolizumab was approved for patients with TMB-H solid tumors, which was the third tumor-agnostic drug approved.

This article is a summary of the part describing TMB-H in “Clinical Practice Guidelines for Tumor-Agnostic Treatments in Adult and Pediatric Patients with Advanced Solid Tumors toward Precision Medicine (in Japanese)”. The part regarding dMMR and NTRK fusion has already been reported elsewhere [4, 5].

The present guidelines provide a guide to diagnosis and treatment and should be utilized in clinical practice according to the recommendation levels described and by adjusting them for individual patients. They are expected to contribute to improving treatment outcomes in patients with solid cancer by utilizing them to perform appropriate tests and treatments on appropriate patients at appropriate timing.

## Materials and methods

The current guidelines systematically describe the items to be considered when treating patients with TMB-H solid tumors, including the timing and methods of testing TMB score and the positioning of immunotherapy. In the clinical setting in Japan, if appropriate tests are performed on appropriate patients and the patients receive appropriate treatment at appropriate timing based on the recommended levels described in the present guidelines, treatment outcomes in patients with solid tumors are expected to be improved.

In the preparation of these guidelines, clinical questions (CQs) were set, and regarding evidence that provides the basis for the answers to those questions, the literature was collected by hand searches and subjected to a systematic review. In setting the CQs, the working group of the Clinical Practice Guidelines for Tumor-Agnostic Genomic Medicine in Adult and Pediatric Patients with Advanced Solid Tumors (3rd edition) prepared draft CQs and decided which ones would be included in the guidelines.

Keywords related to each CQ were selected and sent to the Japan Medical Library Association, which generated queries used to perform comprehensive literature searches. The PubMed, Ichushi Web, and Cochrane Library databases were used in the searches. Important reports by various academic societies also were collected by hand searches and used in the guidelines. Primary and secondary screenings and systematic reviews were performed by persons in charge (SM/YN) of the working group of the Clinical Practice Guidelines for Tumor-Agnostic Genomic Medicine in Adult and Pediatric Patients with Advanced Solid Tumors (3rd edition). The recommendation levels specified for the CQs were determined by voting by the committee members (Table 1). The levels, which were determined based on factors such as the strength of the evidence and the expected benefits and disadvantages for patients, are as follows: strongly recommended (SR), recommended (R), expert consensus opinion (ECO), and not recommended (NR). The status of regulatory approval and insurance coverage in Japan for the treatments (including indications for testing and treatment) was not considered during the voting, but was indicated in the remarks section as needed. The overall assessments based on voting were as follows: (1) SR if ≥ 70% of the votes were for SR; (2) R if criterion (1) was not met and SR votes + R votes accounted for ≥ 70% of the total; (3) ECO if criteria (1) and (2) were not met and SR votes + R votes + ECO votes accounted for ≥ 70% of the total; and (4) NR if NR accounted for ≥ 50% of the total regardless of whether criteria (1), (2), or (3) were met. If all of the criteria (1)–(4) were not met, the assessment was "no recommendation level."

**Table 1:** Degrees of recommendation and decision criteria

Degree of recommendation	Decision criteria
Strongly recommended [SR]	There is sufficient evidence and the benefits of testing outweigh the losses for patients
Recommended [R]	There is certain evidence, considering the balance between benefits and losses for patients
Expert consensus opinion [ECO]	A certain consensus has been obtained, although evidence and information that show patient benefits cannot be said to be sufficient
Not recommended [NR]	There is evidence against efficacy or for adverse outcome, generally not recommended

The recommendations for the CQs include recommendations that are not currently based on strong evidence. As new evidence accumulates, the information and recommendations in these guidelines may change significantly. Although these guidelines will be updated as appropriate, in using a drug clinically, the latest medical information should be reviewed, and every effort made to ensure the drug is used properly.

## Results

### Solid tumors with a high tumor mutation burden (TMB-H)

A characteristic of cancer cells is that they have more genetic mutations than normal cells due to external factors such as exposure to ultraviolet light or smoking, therapeutic interventions such as temozolomide administration, or inborn or acquired genetic causes related to the DNA repair mechanism [6, 7]. The tumor mutation burden (TMB) refers to the quantity of somatic mutations in cancer cells and is expressed in the unit “mut/Mb,” which represents mutations per 1 million bases (1 megabase, or Mb). In preclinical studies, it was found that new peptides produced as a result of non-synonymous mutations, among the passenger gene mutations in cancer cells, are presented as neoantigens by the major histocompatibility complex (MHC) of the surface of antigen-presenting cells and may be recognized as non-self by infiltrating immune cells [8, 9]. Next-generation sequencing technology and calculation methods for predicting antigen peptide presentation by MHC have been developed, and the presence of neoantigens recognized by T cells has been reported in high-TMB mouse tumors, which are similar to high-TMB human tumors [9]. Moreover, immunogenicity associated with increased TMB has been confirmed in nonclinical studies, suggesting that those biological characteristics are applicable across cancer types [10, 11]. Furthermore, a review by Schumacher and Schreiber suggested that in tumors with somatic mutations in excess of 10 mut/Mb (equivalent to 150 non-synonymous mutations), neoantigens that are recognized by the immune system may be produced [12].

### TMB testing

**Fig. 1:**
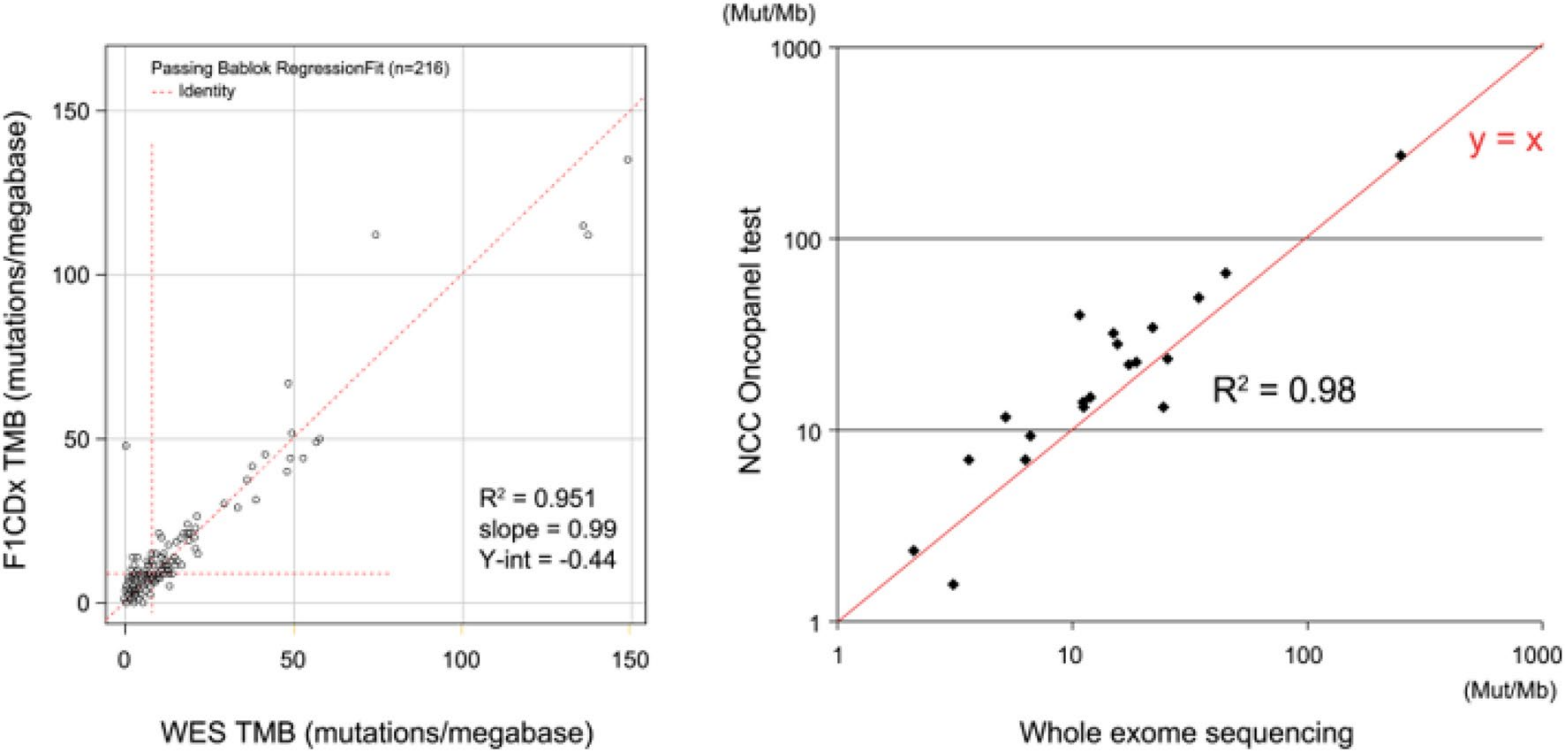
Comparison of tumor mutation burden measured by whole exome sequencing versus comprehensive genomic profiling [13, 18]. Comparison of tumor mutation burden measured by whole exome sequencing versus comprehensive genomic profiling (left: FoundationOne CDx, right: NCC Oncopanel). The line *y* = *x* is plotted in red

TMB has previously been evaluated by whole genome sequencing (WGS) or whole exome sequencing (WES). However, target sequencing panels (gene panel tests) have also recently been found to enable TMB to be assayed with high sensitivity [13–16]. Because TMB scores obtained by gene panel tests in which genome sequencing of a 1.1 Mb TMB analysis region is performed correlate with WES TMB scores, these panel tests can measure TMB accurately. However, with a region of ≤ 0.5 Mb, a lower correlation has been reported [13]. The algorithms used in calculating the TMB value (TMB score) are designed to be optimal for each gene panel. This is problematic because they are the intellectual property of each panel and therefore not openly disclosed, resulting in variability (Table 2) [17]. TMB harmonization is currently being pursued in a TMB harmonization project led by the Friends of Cancer Research (FoCR). FoCR verifies the correlations between TMB scores calculated based on each gene panel test and WES TMB scores. Although there is variability depending on the type of cancer, good correlations have been reported (Spearman’s correlation coefficients of 0.79–0.88). In Japan, testing can be performed as part of comprehensive genomic profiling under the national health insurance coverage. TMB measured by the FoundationOne^®^ CDx assay was found to be highly correlated with WES TMB [10]. A strong correlation was also reported for the NCC Oncopanel [18] (Fig. 1). In the future, FoCR plans to retrospectively analyze the clinical specimens of patients administered immune checkpoint inhibitors in clinical studies with the aim of making TMB testing available in clinical setting.

**Table 2:** Description of the NGS panels [17]

Laboratory	Panel name	#genes	Total region covered (Mb)	TMB region covered (Mb)	Type of exonic mutations included in TMB estimation
ACT Genomics	ACTOnco	440	1.8	1.12	Non-synonymous, synonymous
AstraZeneca	AZ600	607	1.72	1.72	Non-synonymous, synonymous
Caris	SureSelect XT	592	1.6	1.4	Non-synonymous
Foundation Medicine	FoudationOne CDx	324	2.2	0.8	Non-synonymous, synonymous
Guardant Health	GuardantOMNI	500	2.15	1	Non-synonymous, synonymous
Illumina	TSO500	523	1.97	1.33	Non-synonymous, synonymous
Memorial Sloan Kettering	MSK-IMPACT	468	1.53	1.14	Non-synonymous
NeoGenomics	NeoTYPE Discoert Profile for Solid Tumors	372	1.1	1.03	Non-synonymous, synonymous
Personal genome Diagnostics	PGDx elio tissue complete	507	2.2	1.33	Non-synonymous, synonymous
QIAGEN	QIAseq TMB panel	486	1.33	1.33	Non-synonymous, synonymous
Thermo Fisher Scientific	Oncomine Tumor Mutation Load Assay	409	1.7	1.2	Non-synonymous
Sysmex	OncoGUide NCC Oncopanel	124	1.42	1.42	Non-synonymous, synonymous

Pembrolizumab was found to be effective in TMB-H solid tumors in the phase II KEYNOTE-158 study, which used biomarkers to evaluate pembrolizumab efficacy in patients with unresectable advanced or recurrent solid tumors who were refractory or intolerant to prior treatment [18]. In this study, patients with TMB-H ≥ 10 mut/Mb, as analyzed by the FoundationOne^®^ CDx assay, were defined as being TMB-H patients. Based on the results of this study, the FDA approved pembrolizumab for the treatment of TMB-H solid tumors and FoundationOne^®^ CDx as a companion diagnostic for pembrolizumab. In Japan, FoundationOne^®^ CDx was approved on November 15, 2021 to assist in determining whether a drug is indicated for a solid tumor with a high-TMB score.

The conventional method of calculating TMB involves an analysis of tumor tissue. Consequently, in cases such as when a tumor is unresectable and only previously collected surgical specimens can be obtained, TMB analysis using FoundationOne^®^ CDx may not reflect tumor status at the point when systemic treatment is administered. Efforts have therefore been made to evaluate TMB by analyzing circulating tumor DNA (ctDNA) from the blood. As compared with tumor tissue analysis, ctDNA analysis requires less time [19] and may detect intratumoral genetic heterogeneity [20].

### Frequency of TMB-H by cancer type

**Fig. 2:**
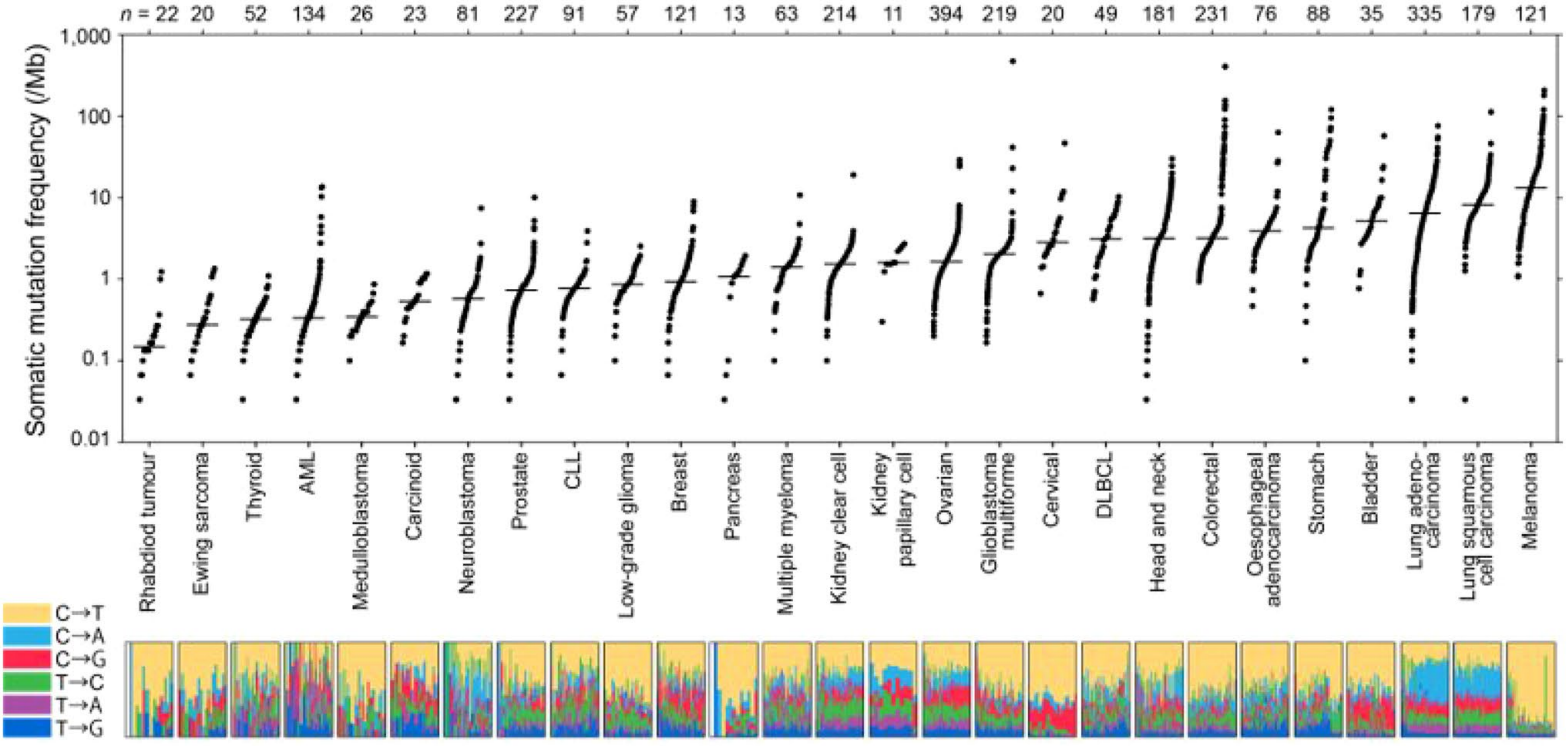
Whole-exome somatic mutation frequencies observed in exomes from tumor–normal pairs [21]. *AML* acute myeloid leukemia, *CLL* chronic lymphocytic leukemia, *DLBCL* diffuse large B-cell lymphoma. Each dot corresponds to a tumor–normal pair, with vertical position indicating the total frequency of somatic mutations in the exome. Mutation frequencies vary more than 1,000-fold between lowest and highest across different cancers and also within several tumor types. The bottom panel shows the relative proportions of the six different possible base-pair substitutions, as indicated in the legend on the left

The frequency of somatic mutations by cancer type is indicated in Fig. 2. The frequency varies widely depending on the cancer type, ranging from more than 100 mut/Mb (e.g., melanoma, lung squamous cell carcinoma/lung adenocarcinoma) to a low 0.1 mut/Mb. Even within the same cancer type, differences of 1000-fold or more are seen [21].

**Fig. 3:**
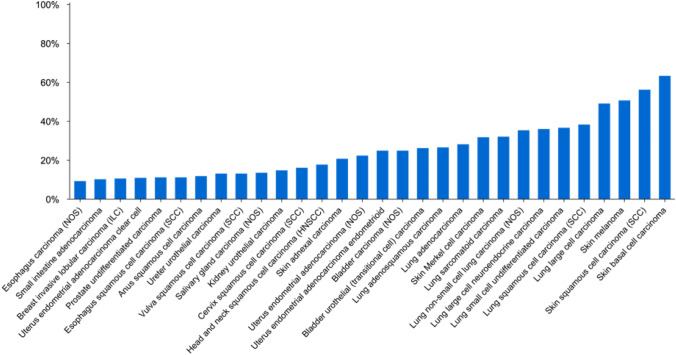
Prevalence of solid tumors with TMB ≥ 10 mut/Mb [22]. *NOS* not otherwise specified. Analysis of top 30 solid tumor types selected from 104,814 total cases sorted by percent of cases with TMB ≥ 10 mut/Mb according to the Foundation Medicine database

The incidence of cancers with TMB scores ≥ 10 mut/Mb as determined using the FoundationOne^®^ CDx assay (see “TMB testing” for more information) was reported in a review by Chan et al. (Fig. 3) [22]. The percentage of the top 30 types of cancer with TMB scores ≥ 10 mut/Mb ranged from approximately 10% to 60% and represented 13.3% of solid tumors as a whole. At a joint meeting of the Japanese Society of Medical Oncology (JSMO), European Society for Medical Oncology (ESMO), American Society of Clinical Oncology (ASCO), and Taiwan Oncology Society (TOS) organized by the Japan Society of Clinical Oncology (JSCO), the incidence of TMB-H tumors in the FoundationOne database was reported using a TMB-H cutoff of TMB ≥ 20 mut/Mb (Table 3) [23]. The percentages in the 30 cancer types with the highest incidences ranged from 0.93 to 54.60%. TMB-H solid tumors have been reported to have a poor prognosis [24].

**Table 3:** Prevalence of high-TMB status in common adult tumors [23]

Group ontology	No samples	No TMB-H	% TMB-H	Lower 95% CI (%)	Upper 95% CI (%)
Skin	934	510	54.60	51.35	57.83
Melanoma	5602	1842	32.88	31.65	34.13
Diffuse large B-cell lymphoma	785	148	18.85	16.17	21.77
Underspecified	740	97	13.11	10.76	15.75
Endometrial	6112	730	11.94	11.14	12.78
Non-small cell lung cancer	39,746	4559	11.47	11.16	11.79
Bladder	3425	389	11.36	10.31	12.47
Unknown primary carcinoma	10,636	925	8.70	8.17	9.25
Head and neck	3145	250	7.95	7.03	8.95
Salivary gland	962	68	7.07	5.53	8.88
Small cell lung cancer	2470	166	6.72	5.76	7.78
Cervix	1678	103	6.14	5.04	7.40
Small intestine	1027	63	6.13	4.75	7.78
Unknown primary-neuro	1386	79	5.70	4.54	7.05
Colorectal	24,747	1263	5.10	4.83	5.39
Stomach	3558	173	4.86	4.18	5.62
Anus	623	24	3.85	2.48	5.68
Glioma	6395	238	3.72	3.27	4.22
Uterus	1080	40	3.70	2.66	5.01
Gastrointestinal-neuro	602	21	3.49	2.17	5.28
Prostate	7222	220	3.05	2.66	3.47
Soft tissue sarcoma	4164	115	2.76	2.29	3.31
Breast	19,024	496	2.61	2.39	2.84
Esophagus	5329	135	2.53	2.13	2.99
Biliary	2182	45	2.06	1.51	2.75
Appendix	959	19	1.98	1.20	3.08
Endocrine-neuro	947	16	1.69	0.97	2.73
Kidney	3419	51	1.49	1.11	1.96
Cholangiocarcinoma	3905	45	1.15	0.84	1.54
Plasma cell neoplasm	1606	15	0.93	0.52	1.54

Effort also has been made to evaluate TMB by cancer type using ctDNA analysis. Foundation Medicine Inc. developed an assay to evaluate blood TMB (bTMB) by analyzing blood ctDNA. Pretherapy baseline bTMB in blood specimens was evaluated in the prospective POPLAR and OAK studies, which evaluated the efficacy of atezolizumab as compared with that of docetaxel in non-small cell lung cancer [25]. Tissue TMB (tTMB) also was analyzed at the same time in these studies using tumor tissue, and the sensitivity and specificity of bTMB as compared with tTMB were found to be 64% and 88%, respectively. FoundationOne^®^ Liquid CDx developed by Foundation Medicine Inc. was approved in Japan in March 2021 for comprehensive genomic profiling of solid tumors using blood specimens.

**Fig. 4:**
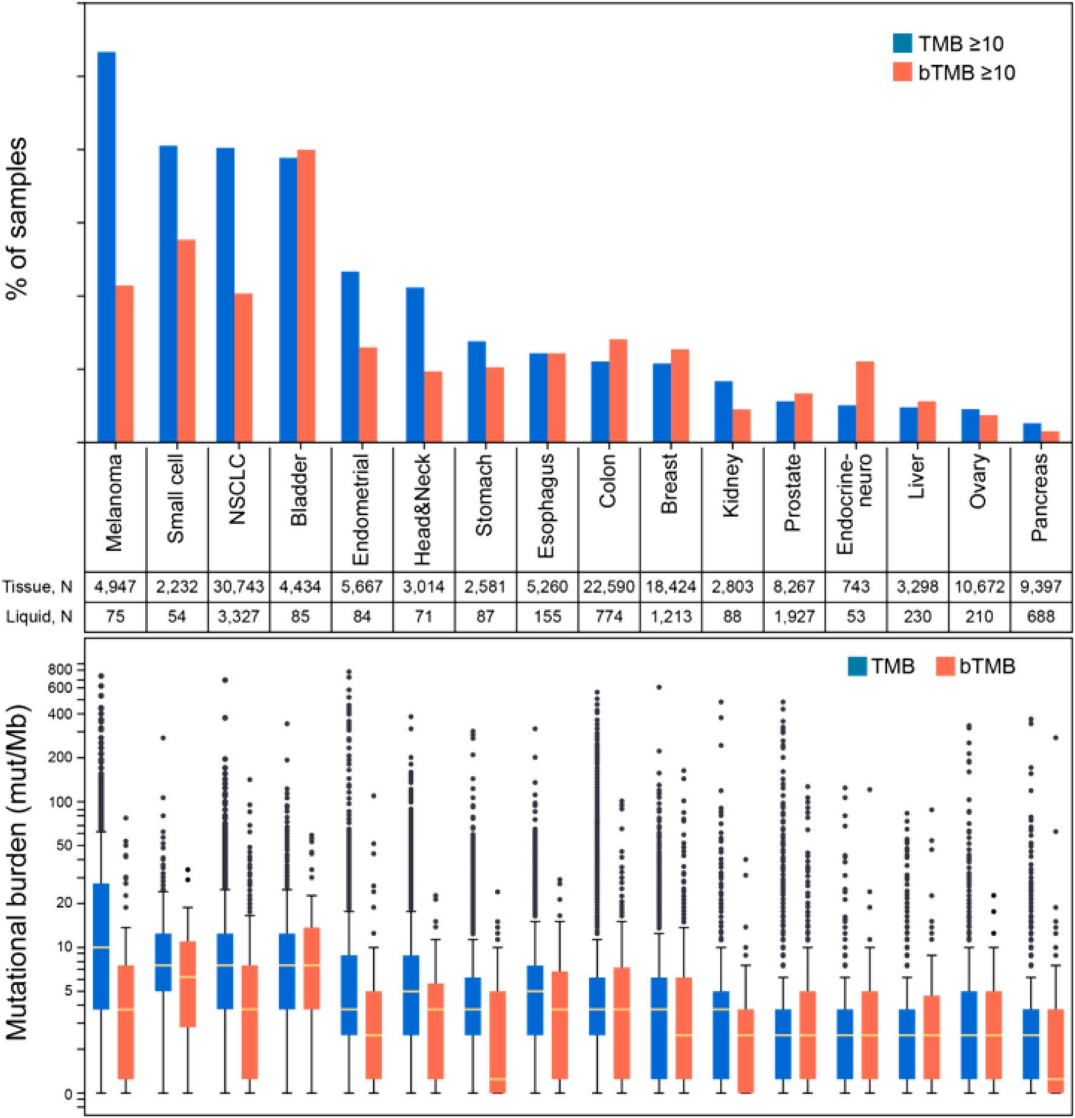
Prevalence of solid tumors with TMB ≥ 10 mut/Mb and bTMB ≥ 10 mut/Mb [25]. *NSCLC* non-small cell lung cancer. Pan-cancer, TMB ≥ 10 was detected in 19% of tissue cases (31,272/167,332) and was common in melanoma (53%), small cell (41%), NSCLC (40%), bladder (39%), and endometrium (23%). bTMB ≥ 10 was detected in 13% of liquid biopsies (1195/9312); prevalence by cancer type was correlated with prevalence of elevated tissue TMB (*r* = 0.81)

The incidences of tTMB-H (≥ 10 mut/Mb), analyzed in tumor tissue using the FoundationOne^®^ CDx assay, and bTMB-H (≥ 10 mut/Mb), analyzed in blood using the FoundationOne^®^ Liquid CDx assay, have been reported by cancer type (Fig. 4) [26]. Sixteen cancer types in 167,332 patients were analyzed. The incidence of tTMB-H was 19%, and the cancer types with the highest incidences of tTMB-H were, in descending order of incidence, malignant melanoma (53%), small cell lung cancer (41%), non-small cell lung cancer (40%), bladder cancer (39%), and endometrial cancer (23%). In the bTMB analysis, which examined 16 cancer types in 9312 patients, the incidence of bTMB-H was 13%. The prevalence by cancer type was correlated with prevalence of elevated tissue TMB (r = 0.81). While there are some reports of high correlation in lung cancer, there are also reports of low correlation and concordance rates in gastrointestinal cancer, and differences by metastatic organ have also been reported [27, 28]. Further investigation of bTMB is needed.

### Efficacy of anti-PD-1/PD-L1 antibody drugs against TMB-H solid tumors

**Fig. 5:**
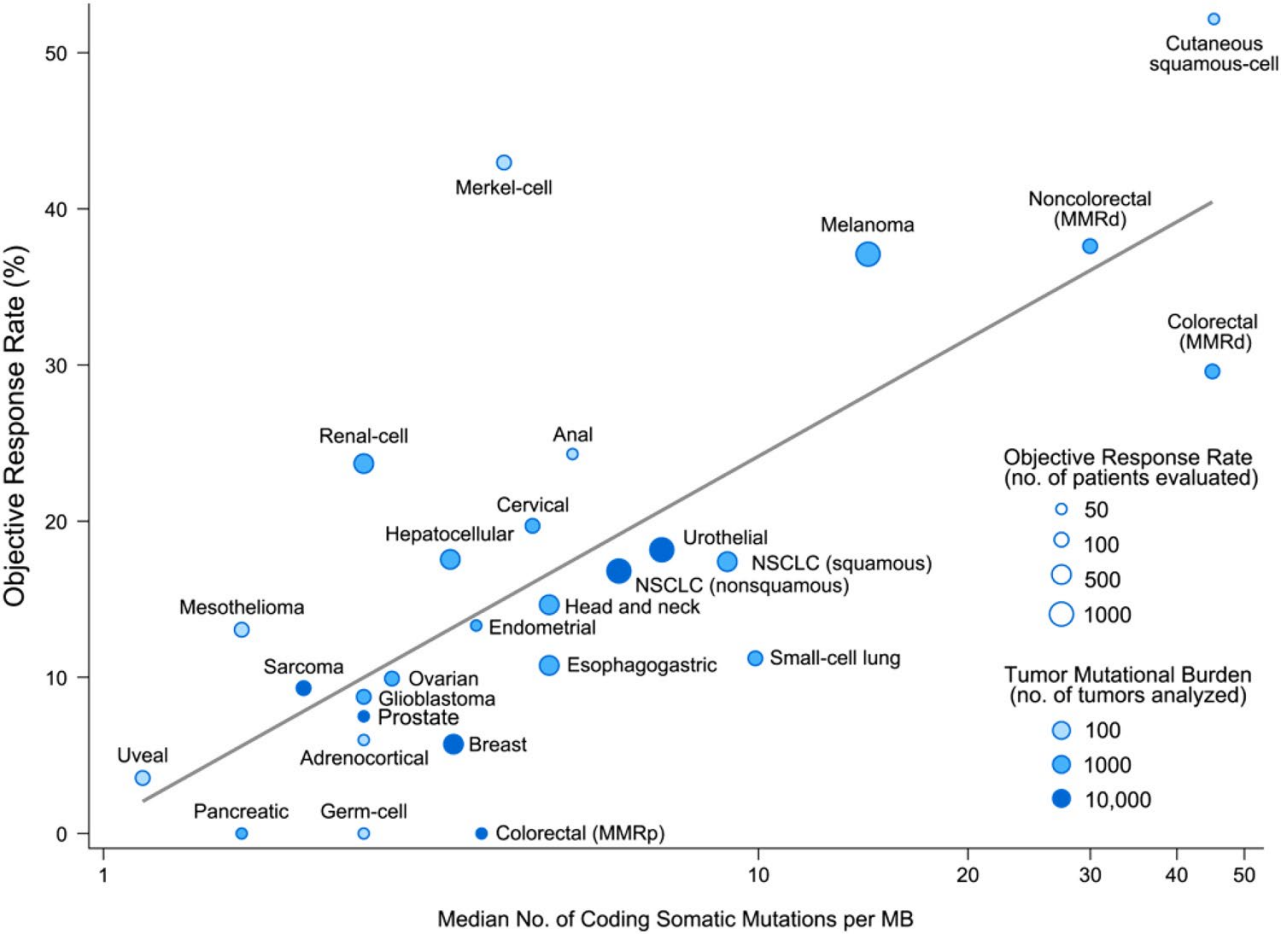
Correlation between TMB and ORR with anti-PD-1/PD-L1 therapy in various cancer types [32]. *MMRd* mismatch repair deficient, *NSCLC* non-small cell lung cancer. Shown is the median number of coding somatic mutations per megabase (MB) of DNA in 27 tumor types or subtypes among patients who received inhibitors of programmed death 1 (PD-1) protein or its ligand (PD-L1), as described in published studies for which data regarding the objective response rate are available. The number of patients who were evaluated for the objective response rate is shown for each tumor type (size of the circle), along with the number of tumor samples that were analyzed to calculate the tumor mutational burden (degree of shading of the circle). Data on the x axis are shown on a logarithmic scale

In preclinical studies, novel peptides produced by amino acid substitutions resulting from DNA mutations among the passenger gene mutations of cancer cells were presented as neoantigens, causing an antitumor immune response [8, 9]. Mouse tumors with high TMB have been reported to have neoantigens that are recognized by T cells [9]. Moreover, immunogenicity associated with increased TMB has been confirmed in nonclinical studies [10, 11, 29], suggesting that an increase in neoantigens resulting from increased TMB in tumor cells may promote tumor recognition by T cells. Immune checkpoint inhibitors are therefore likely to have an antitumor effect in TMB-H solid tumors by facilitating T-cell activation. The KEYNOTE-028 study was a phase Ib study that examined the safety and efficacy of pembrolizumab in advanced solid tumors positive for PD-L1 expression. As an exploratory endpoint, the study examined the relationship between TMB and PD-L1. WES TMB was analyzed for 16 cancer types in 77 patients (only 1 of whom was MSI-H), and a stronger tumor regression effect and prolongation of PFS were seen in patients with high TMB [30]. TMB was investigated using the MSK-IMPACT platform in 1662 patients who received immune checkpoint inhibitor monotherapy or combination therapy at the Memorial Sloan Kettering Cancer Center in the United States. A comparison of patients with the highest 20% of TMB scores and the other patients by cancer type showed that OS was significantly longer in the former group (HR: 0.52; *p* = 1.6 × 10^−6^) [31]. In addition to these studies, many others have also found TMB to be a useful factor for predicting the efficacy of immune checkpoint inhibitors. When the objective response rate (ORR) seen with immune checkpoint inhibitor monotherapy (anti-PD-1 or anti-PD-L1 antibody) was plotted against the median TMB for 27 cancer types, a significant correlation between ORR and TMB was observed (Fig. 5) [32].

The KEYNOTE-158 study was a multicenter, non-randomized, open-label, multicohort phase II study that evaluated the efficacy and safety of pembrolizumab in patients with unresectable or metastatic solid tumors who were refractory or intolerant to prior treatment. The study evaluated various biomarkers that predict pembrolizumab efficacy in a variety of cancer types. TMB was designated in advance as an exploratory biomarker and was analyzed retrospectively using the FoundationOne^®^ CDx assay. As a post-marketing requirement for FDA approval in the United States, Group M was subsequently added as prospectively enrolled cohort of patients with TMB-H solid tumors. The primary endpoint of the study was ORR, and the secondary endpoints were the duration of response, progression-free survival (PFS), and overall survival (OS). TMB data were obtained for 790 of the 1050 patients in the efficacy analysis set. Using a TMB-H cutoff of ≥ 10 mut/Mb, 102 patients were classified as TMB-H and 688 as TMB-Low (TMB-L, < 10 mut/Mb). Pembrolizumab showed a higher ORR in the TMB-H group than in the TMB-L group (29% vs. 6%). With MSI-H patients and those whose MSI status was unknown excluded, the ORR in 81 patients in the TMB-H group was 28%, which was comparable. PD-L1 expression was also evaluated in this study. No correlation was seen between TMB score and PD-L1 expression (combined positive score: CPS), and ORR was 35% for PD-L1-positive patients (CPS ≥ 1) in the TMB-H group and 21% for PD-L1-negative patients (CPS < 1) in this group [33]. Based on the results of this study, the FDA approved pembrolizumab for the treatment of TMB-H solid tumors.

The Targeted Agent and Profiling Utilization Registry (TAPUR) study, which was conducted by ASCO, was a phase II basket study that evaluated the antitumor effects of approved targeted drugs in specific genomic alterations. The results for a TMB-H cohort in the study also were reported. An examination of 27 patients with colorectal cancer with TMB ≥ 9 (25 MSS patients, 2 patients with unknown microsatellite status) showed an antitumor effect, with an ORR of 11% (95% CI 2–29%) median PFS of 9.3 weeks (95% CI 7.3–16.1), and median OS of 51.9 weeks (95% CI 18.7–NR) [34]. A similar examination was conducted in breast cancer with TMB ≥ 9 and an antitumor effect was also seen, with the ORR of 37% (95% CI 21–50%), median PFS of 10.6 weeks (95% CI 7.7–21.1), and median OS of 30.6 weeks (95% CI 18.3–103.3) [35].

Even since the FDA approved pembrolizumab for TMB-H solid tumors, debate has continued regarding the TMB-H cutoff and differences in efficacy in each type of cancer. Immune checkpoint inhibitors showed a strong antitumor effect (ORR: 39.8%; 95% CI 34.9–44.8) in TMB-H tumors for cancer types in which tissue invasive CD8 T-cell levels showed a positive correlation with neoantigen levels, such as malignant melanoma, lung cancer, and bladder cancer. The ORR in TMB-H tumors was significantly higher than in TMB-L tumors [odds ratio (OR): 4.1; 95% CI 2.9–5.8; *p* < 2 × 10^−16^]. However, in cancer types for which there was no correlation between CD8 T-cell levels and neoantigen levels, such as breast cancer, prostate cancer, and glioma, the ORR of immune checkpoint inhibitors in TMB-H tumors was 15.3% (95% CI 9.2–23.4; p = 0.95), which was significantly lower than in TMB-L tumors (OR: 0.46; 95% CI 0.24–0.88; *p* = 0.02) [36]. This suggested that, depending on the cancer type, it may not be possible to predict the efficacy of immune checkpoint inhibitors based on TMB. It has also been suggested that the optimal TMB cutoff may differ depending on cancer type [37]. In gliomas, temozolomide therapy results in an increase in TMB, although the mechanism of that change is unknown. However, in an examination of the efficacy of an immune checkpoint inhibitor in 11 patients with TMB-H and dMMR gliomas that included such patients (5 untreated and 6 posttreatment patients), the best therapeutic effect was disease progression in 82%, with no significant difference seen as compared with TMB-L gliomas [38]. These findings indicate a need for further investigation regarding the optimal method of measuring TMB and the TMB cutoff for each cancer type.

Efforts have also been made to evaluate TMB by ctDNA analysis. In 69 patients with solid tumors who were administered an immune checkpoint inhibitor, ctDNA from the blood was analyzed using the Guardant360 assay, a ctDNA testing method. The results showed that PFS was significantly longer in patients with more than 3 variants of unknown significance (VUS) [39]. Moreover, in the OAK and POPLAR studies, which examined the superiority of atezolizumab versus docetaxel in non-small cell lung cancer, ctDNA analysis using the FoundationOne bTMB assay showed that atezolizumab efficacy was greatest in patients whose bTMB score was ≥ 16 [25].

## CQs

The following requirements have been prepared regarding the TMB testing performed to select patients who are likely to benefit from PD-1/PD-L1 inhibitors and the administration of them. The clinical recommendations propose the following 7 requirements in 3 CQs regarding the TMB testing performed to select patients who are likely to benefit from anti-PD-1/PD-L1 antibody drugs.

1. For patients with solid tumors who are undergoing standard drug therapy or for whom standard treatment is difficult to administer, other than those for which immune checkpoint inhibitors can be used clinically irrespective of the TMB score, TMB testing is recommended to determine whether immune checkpoint inhibitors are indicated.
2. For patients with unresectable solid tumors for which immune checkpoint inhibitors can already be used clinically irrespective of the TMB score, TMB testing should be considered to determine whether immune checkpoint inhibitors are indicated.
3. For patients with solid tumors that are curable with local treatment, TMB testing is not recommended to determine whether immune checkpoint inhibitors are indicated.
4. For patients with unresectable solid tumors for which an immune checkpoint inhibitor has already been used, TMB testing is not recommended to determine again whether immune checkpoint inhibitors are indicated.
5. As TMB testing to determine whether immune checkpoint inhibitors are indicated, an NGS test whose analytical validity has been established (by receiving regulatory approval, etc.) is recommended.
6. For unresectable/metastatic/recurrent solid tumors with TMB-H, the use of immune checkpoint inhibitors is recommended.
7. The use of immune checkpoint inhibitors is recommended for unresectable/metastatic/recurrent solid tumors that have progressed after chemotherapy.

Please keep in mind that these clinical recommendations will be revised in a timely manner, along with continuously and steadily advancing cancer treatment and new knowledge on biomarkers.

We will explain each CQ in detail.

## CQ1: Targets of TMB testing

PubMed was searched using the following queries: “Mutation and Tumor Burden or burden * or TMB,” “neoplasm,” and “tested or diagnos* or detect*.” The same queries were used to search Cochrane Library. For the search period from January 1980 to January 2021, 585 articles were extracted from PubMed and 26 from Cochrane Library. In the primary screening, 233 articles were extracted, and 208 were extracted in the secondary screening. A qualitative systematic review of these articles was then performed.
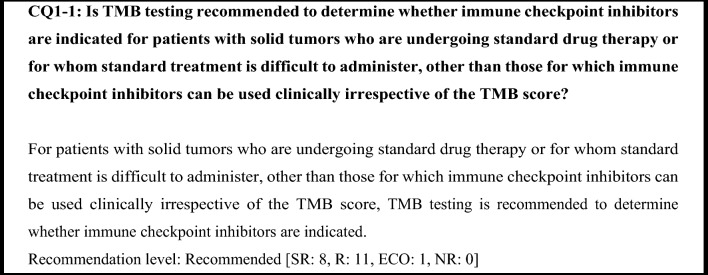


The KEYNOTE-158 study examined the efficacy of pembrolizumab in advanced or recurrent solid tumors that progressed after chemotherapy. The TMB score was measured using the FoundationOne^®^ CDx assay, and ≥ 10 mut/Mb was used as the TMB-H cutoff. The results showed that the ORR of pembrolizumab was higher in the TMB-H group than in the TMB-L group (29% vs. 6%) [17]. Based on the results of this study, the United States FDA granted the expedited approval of pembrolizumab for unresectable or metastatic TMB-H (≥ 10 mut/Mb) solid tumors on June 16, 2020. In addition, FoundationOne^®^ CDx was approved as a pembrolizumab companion diagnostic. TMB is therefore considered a valid biomarker for immune checkpoint inhibitor use and is also recommended in Japan.
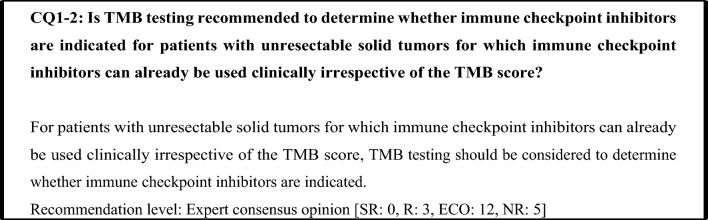


TMB testing is generally considered unnecessary in solid tumors for which immune checkpoint inhibitors can be used irrespective of the TMB score, because determining whether such use is indicated does not depend on the TMB score. However, in solid tumors for which determination regarding whether immune checkpoint inhibitors are indicated is based on PD-L1 expression or a biomarker such as dMMR, an immune checkpoint inhibitor is likely to be effective if the biomarker test was negative. In the KEYNOTE-158 study, ORR was 28% and efficacy was seen regardless of PD-L1 expression (ORR was 35% in PD-L1-positive patients and 21% in PD-L1-negative patients) in the patients with TMB-H after the exclusion of MSI-H patients and patients whose MSI status was unknown [33]. Based on the above findings, in solid tumors for which determination regarding whether immune checkpoint inhibitors are indicated is based on a biomarker, TMB testing is recommended if the biomarker test was negative.
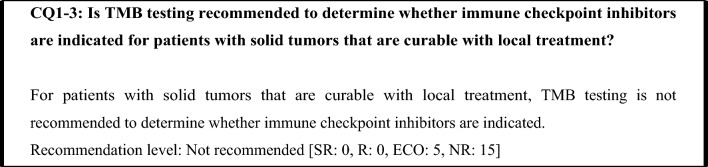


In malignant melanoma, an anti-PD-1 antibody drug has been shown to be effective as postoperative adjuvant therapy and has been approved (KEYNOTE-054 study [40], ONO-4538–21 study [41]). In the multicenter, double-blind, randomized, placebo-controlled phase III PACIFIC study, an anti-PD-L1 antibody drug was administered sequentially in patients with unresectable, locally advanced (Stage III) non-small cell lung cancer that did not progress following curative concurrent chemoradiotherapy (CRT) using a platinum drug. Based on the results of that study, anti-PD-L1 antibody therapy received regulatory approval [42]. In the Checkmate-577 study, the efficacy of nivolumab as postoperative adjuvant therapy was shown in Stage II/III esophageal and gastroesophageal junction cancer that was resected after neoadjuvant chemoradiotherapy [43]. However, because no differences in efficacy according to TMB score were reported in these studies, pretreatment TMB testing is generally unnecessary. Moreover, because the efficacy of immune checkpoint inhibitors as perioperative therapy has not been established for other solid tumors, TMB testing for treatment selection is generally unnecessary for such cancers if the cancer can be cured with local treatment. Based on the above considerations, TMB testing is currently not recommended to determine whether immune checkpoint inhibitors are indicated for patients with solid tumors that are not locally advanced and have not metastasized.
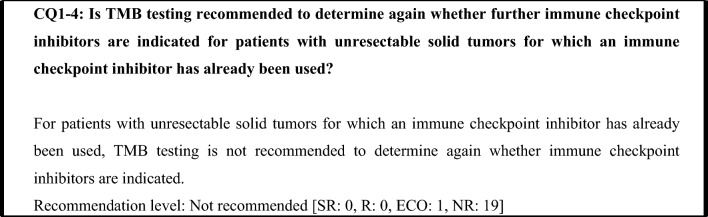


Immune checkpoint inhibitors have been approved for use in some solid tumors irrespective of the TMB score. The effectiveness of using a different immune checkpoint inhibitor when one has already been administered has not been demonstrated. Therefore, TMB testing is not recommended for the purpose of using an immune checkpoint inhibitor in patients with solid tumors for which an immune checkpoint inhibitor has already been used.

## CQ2: Testing methods of TMB

PubMed was searched using the following queries: "Mutation and Tumor Burden or burden * or TMB," and "next-generation sequencing or NGS or Whole-exome sequencing or WES." The same queries were used to search Cochrane Library. For the search period from January 1980 to January 2021, 387 articles were extracted from PubMed and 22 from Cochrane Library. In the primary screening, 215 articles were extracted, and 204 were extracted in the secondary screening. A qualitative systematic review of these articles was then performed.
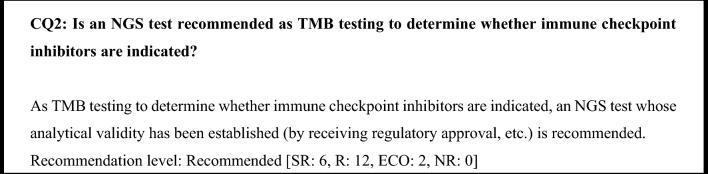


FoundationOne^®^ CDx was approved in Japan on December 27, 2018 for the purpose of obtaining comprehensive genomic profiles of tumor tissue in patients with solid tumors and for the purpose of detecting somatic gene alterations in order to determine whether some molecularly targeted drugs are indicated in such patients. FoundationOne^®^ CDx also includes TMB score information. The KEYNOTE-158 study measured TMB scores using the FoundationOne^®^ CDx assay and examined the efficacy of pembrolizumab in advanced or recurrent solid tumors that progressed after chemotherapy, with a TMB-H cutoff of ≥ 10 mut/Mb. The results showed that the ORR of pembrolizumab was higher in the TMB-H group than in the TMB-L group [17]. Based on the results of this study, the FDA granted expedited approval of pembrolizumab for unresectable or metastatic TMB-H (≥ 10 mut/Mb) solid tumors on June 16, 2020. In addition, FoundationOne^®^ CDx was approved as a pembrolizumab companion diagnostic. In Japan, FoundationOne^®^ CDx was approved on November 15, 2021 to assist in determining whether drugs for solid tumors with high-TMB scores are indicated.

In addition to FoundationOne^®^ CDx, OncoGuide™ NCC Oncopanel System was approved in Japan as a comprehensive genomic profiling test for tumor tissue in patients with solid tumors. As with the FoundationOne^®^ CDx assay, a strong correlation with WES has been reported for this test [15], indicating that it can predict the therapeutic benefit of an immune checkpoint inhibitor. However, as of June 2021, there had been no reports of studies examining the efficacy of an immune checkpoint inhibitor using the OncoGuide™ NCC Oncopanel System. The algorithm used to calculate TMB scores varies depending on the gene panel used, and attention must therefore be paid to the resulting variability. The FoCR is currently performing a retrospective analysis of clinical specimens from patients administered immune checkpoint inhibitors in clinical studies, and it is anticipated that uniform TMB scores obtained with different gene panels will be available in clinical setting.

In addition, the FoundationOne^®^ Liquid CDx Cancer Genome Profile was approved as a comprehensive genomic profiling test for solid tumors using blood specimens on March 22, 2021, and an application for marketing approval of Guardant360CDx was filed on January 28, 2021. Thus, opportunities to perform measurements in clinical practice are expected to increase. The OAK and POPLAR studies, which examined the superiority of atezolizumab versus docetaxel in non-small cell lung cancer, analyzed blood specimens using a bTMB assay and found that atezolizumab efficacy was greater in patients with bTMB scores ≥ 16 [25]. It is anticipated that the efficacy of atezolizumab is verified in other cancer types.

Based on the above findings, an NGS test whose analytical validity has been established using tissue is recommended as TMB testing to determine whether immune checkpoint inhibitors are indicated.

## CQ3: Treatment for TMB-H cancer

PubMed was searched using the following queries: "Mutation and Tumor Burden or burden * or TMB," "PD-1 or PD-L1 *," and "treat*." The same queries were used to search Cochrane Library. For the search period from January 1980 to January 2021, 323 articles were extracted from PubMed and 10 from Cochrane Library. In the primary screening, 74 articles were extracted, and 71 were extracted in the secondary screening. A qualitative systematic review of these articles was then performed.
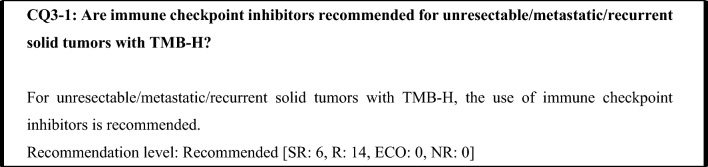


The KEYNOTE-158 study measured TMB scores using the FoundationOne^®^ CDx assay and examined the efficacy of pembrolizumab in advanced or recurrent solid tumors that progressed after chemotherapy, with a TMB-H cutoff of ≥ 10 mut/Mb. The results showed that the pembrolizumab ORR was higher in the TMB-H group than in the TMB-L group (29% vs. 6%) [33]. Immune checkpoint inhibitors have been shown to have a tumor-agnostic therapeutic effect in TMB-H tumors. It should be noted, however, that the reported sample sizes have been limited for some cancer types and that immune checkpoint inhibitors have shown no efficacy in some cancer types (see "4 Efficacy of anti-PD-1/PD-L1 antibody drugs against TMB-H solid tumors").
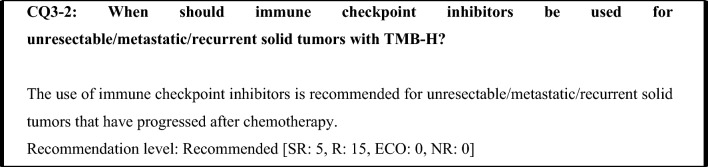


The efficacy of immune checkpoint inhibitors in treating TMB-H solid tumors was shown in the KEYNOTE-158 study, which examined advanced or recurrent solid tumors that progressed after chemotherapy. Therefore, it is currently not the first-line therapy of choice. In view of the turnaround time (TAT) required for TMB testing, it is generally considered preferable to start the first-line therapy established for each organ (standard treatment) without waiting for the results of TMB testing. However, TMB is an important biomarker for investigating subsequent therapy, and testing for it and other biomarkers should therefore be considered at an early stage.

## Conclusion

Immune checkpoint inhibitors significantly prolongs patient survival in many types of cancers; however, significant resistance to this therapeutic modality has been reported. Thus, to identify patients who are more likely to benefit from this therapy, various biomarkers, including TMB, have been examined. However, there are some issues related to administering immune checkpoint inhibitors for TMB-H tumors in the clinical setting. In this guideline, the panel recommends the requirements for performing TMB testing properly to select patients who are likely to benefit from immune checkpoint inhibitors.
